# ChatGPT's performance before and after teaching in mass casualty incident triage

**DOI:** 10.1038/s41598-023-46986-0

**Published:** 2023-11-21

**Authors:** Rick Kye Gan, Helal Uddin, Ann Zee Gan, Ying Ying Yew, Pedro Arcos González

**Affiliations:** 1 Unit for Research in Emergency and Disaster, Faculty of Medicine and Health Sciences, University of Oviedo, 33006 Oviedo, Spain; 2 Department of Global Public Health, Karolinska Institute, 17177 Solna, Sweden; 3 Department of Sociology, East West University, Dhaka, 1212 Bangladesh; 4 Tenghilan Health Clinic, 89208 Tuaran, Sabah Malaysia

**Keywords:** Health care, Medical research

## Abstract

Since its initial launching, ChatGPT has gained significant attention from the media, with many claiming that ChatGPT’s arrival is a transformative milestone in the advancement of the AI revolution. Our aim was to assess the performance of ChatGPT before and after teaching the triage of mass casualty incidents by utilizing a validated questionnaire specifically designed for such scenarios. In addition, we compared the triage performance between ChatGPT and medical students. Our cross-sectional study employed a mixed-methods analysis to assess the performance of ChatGPT in mass casualty incident triage, pre- and post-teaching of Simple Triage And Rapid Treatment (START) triage. After teaching the START triage algorithm, ChatGPT scored an overall triage accuracy of 80%, with only 20% of cases being over-triaged. The mean accuracy of medical students on the same questionnaire yielded 64.3%. Qualitative analysis on pre-determined themes on ‘walking-wounded’, ‘respiration’, ‘perfusion’, and ‘mental status’ on ChatGPT showed similar performance in pre- and post-teaching of START triage. Additional themes on ‘disclaimer’, ‘prediction’, ‘management plan’, and ‘assumption’ were identified during the thematic analysis. ChatGPT exhibited promising results in effectively responding to mass casualty incident questionnaires. Nevertheless, additional research is necessary to ensure its safety and efficacy before clinical implementation.

## Introduction

Since its initial unveiling in November 2022, ChatGPT, an artificially-intelligence (AI) chatbot, has attained great curiosity in media and is designated as a transformative milestone in the progresst of the AI revolution^1,2^. ChatGPT has gained recognition for its ability to facilitate information search, provide answers to inquiries, and offer exciting guidance^3,4^. AI-based tools such as ChatGPT possess the potential for transformative implications in the realm of healthcare, ranging from revolutionizing drug discovery to optimizing healthcare management and improving access to care by maximizing the utilization of available resources^5,6^. Hence, the uses of AI in public health, health care system, and medicine are boundless, as many scientific investigations have already been conducted on using AI applications in medicine and clinical settings to face global health challenges^6–9^.

In addition to its applications in the clinical setting, ChatGPT plays a significant role in public health by disseminating crucial information on health-related topics and community health services, addressing queries regarding health promotion, and offering guidance on disease prevention strategies among other functions^10^. Its progressive application extends across all areas of medicine, from primary care to emergency medicine, particularly in different stages of patient management following any disasters^11^. For instance, AI-based decision-making would help to maintain improved flow metrics in the emergency department (ED), optimizing the limited resources^12,13^. Similarly, a recent study also documented that AI can accelerate organizational planning and management and improve diagnosis in ED^12^. However, more knowledge is needed about using AI tools, especially the application of ChatGPT in the context of mass casualty incidents (MCIs) triage and disaster medicine.

Triage is the process of categorizing medical conditions into different groups based on the severity of the victim's condition and the available medical resources to prioritize care effectively^14,15^. Generally, triage for mass casualties or disasters is conducted at the incident scene by prehospital medical responders to undertake the decisions about which victims are treated on the spot and who will be transported immediately to the nearest hospital or other healthcare centers^15,16^. In order to effectively triage after an MCI, triage workers need a consistent and accurate method for determining triage categories. In fact, if triage workers conduct triage prioritization incorrectly and with a poor level of accuracy (over-triage and under-triage), it would adversely affect the disaster mitigation initiatives and outcomes, resulting in a great loss of lives and resources^17^. For example, using a different casualty scenario paper exercise, previous studies documented that the mean accuracy score for triage categories among medical student volunteers was 64.3%^18^, and about 65% among physicians and nurses, whereas 59% accuracy was achieved by paramedics^19,20^. On the other hand, AI and machine learning (ML) models can be used effectively in decision-making and information processing for emergency management and mass casualty prevention^21^. For example, AI-based decision-making models are suitable for the development of emergency response plans^22^. Moreover, another study using MCIs data documented that data-driven AI models effectively reduced the time needed for triage using a wearable device and ensured the feasibility of remote triage^23^.

Hence, to reduce the burden of over and under-triage, we hypothesized that ChatGPT may work as a useful tool for the decision-making process with a higher level of accuracy in triage performance following mass casualties. Although there have been studies exploring the application of AI tools in emergency medicine and ED, there are no studies on how ChatGPT performs in patient prioritization during MCI triage compared to other triage workers (i.e., physicians, nurses, medical students, paramedics, etc.,), which is filled by the current study. Hence, this study aimed to measure the performance of ChatGPT in the triage of MCIs using a validated questionnaire particularly designed for such scenarios. Furthermore, our analysis compared the triage performance of ChatGPT with that of medical students.

## Method

### Study design

The design of our study involved a cross-sectional approach, which incorporated a mixed-methods analysis to evaluate the performance of ChatGPT in mass casualty incident triage before and after the teaching of Simple Triage And Rapid Treatment (START) triage. Firstly, we conducted a quantitative descriptive analysis to assess ChatGPT's overall MCIs triage performance. Next, we compared the accuracy of ChatGPT's performance to that of medical students on the same triage questionnaire. Lastly, the qualitative component of our study involved a detailed exploration of ChatGPT's responses, utilizing thematic and content analysis techniques.

### Setting and participant

We used the OpenAI model of ChatGPT-3.5 March 23 version (OpenAI; San Francisco, CA), a free and open-access AI chatbot^24^. The test was conducted and completed on Mar 31, 2023. The account had not been introduced to any information or knowledge regarding MCIs before the data collection.

### Materials

We applied validated a mass casualty incident triage questionnaire and the medical student’s triage performance result, published by Sapp et al. 2010^18^ with written permission. The triage questionnaire consists of 15 triage scenarios developed by emergency medical services (EMS) Medical Directors and Emergency Faculty who have received direct disaster management training and disaster response experience at the University of North Carolina School of Medicine. The patient scenarios selected for the triage questionnaire were designed to maintain a balanced distribution of triage levels and to ensure that the answers aligned with the START triage criteria.

Each scenario provided comprehensive background information on the patient's age, clinical symptoms, vital signs (such as respiration rate, pulse rate, capillary refill), and mode of arrival at the medical facility. In addition, the development of the triage questionnaire scenario considered a diverse range of medical and traumatic presentations unrelated to sarin gas exposure^18^. As a result, four patient scenarios were triaged as 'Red' (Immediate), four were 'Yellow' (Delayed), four were 'green' (minor), and three were 'black' (deceased). The complete triage questionnaire is in Supplementary Table S1.

### Data collection

Data collection for our study involved three phases, namely:

Initial prompt before teaching START triage

In the first data collection phase, before teaching ChatGPT about the START triage system, we presented an initial prompt to verify ChatGPT's familiarity with the subject. Once we received confirmation, we introduced the triage scenarios, one question at a time, from the mass casualty triage questionnaire (Supplementary Table S1). ChatGPT's responses were documented and recorded in an Excel spreadsheet for subsequent analysis.

Teaching prompt on how to perform START triage

During the second phase, we taught ChatGPT the correct steps of START triage according to START triage guidelines and algorithms^25^. We clarified the medical abbreviations used in the questionnaire, as it is well-known that medical abbreviations could be misleading and dangerous^26,27^, the complete prompt is shown in Supplementary Table S2.

Re-test prompt after teaching START triage

In the final phase, we again re-introduced the triage scenarios from the mass casualty triage questionnaire with one question at a time. Additional prompt of "by using the newly taught START triage, triage this case:" used before every mass casualty triage questionnaire scenario. ChatGPT's responses were documented and recorded in an Excel spreadsheet for subsequent analysis. In all phases, data collection was undertaken within a single chat.

### Data analysis

Firstly, we analyzed the overall triage performance of ChatGPT on the mass casualty triage questionnaire before and after teaching the START triage. Triage performance was categorized into (1) Correct triage, (2) Over-triage, and (3) Under-triage. *Over-triage* is a triage that leads to unnecessary use of resources or overutilization^28^. Examples of over-triage are cases with 'minor,' 'delayed,' or 'deceased' being wrongly triaged as the 'immediate' category. On the other hand, *Under-triage* is defined as a triage that guides to suboptimal care resulting an increased risk of mortality and adverse outcome^28^. Examples of under-triaged are cases with the 'immediate' category being wrongly triaged as 'minor,' 'delayed,' or 'deceased.' Descriptive statistics were employed to determine the percentage of ChatGPT's performance across all three triage performance categories.

Next, we analyzed ChatGPT's response in depth by using pre-determined themes on (1) Walking wounded, (2) Respiration, (3) Perfusion, and (4) Mental status. This task was undertaken by two authors, AZG and YYY. The performance of each theme was categorized as correct or incorrect using START adult triage guidelines and algorithm as reference^25^. In accordance with the questionnaire, statements that accurately depicted the patient's scenario and triage decision following the START triage algorithm were considered correct. At the same time, those that did not meet these criteria were classified as incorrect. Finally, the task of exploring a new theme derived from the data was undertaken by the first author RKG. Additional four themes were identified (1) Disclaimer, (2) Patient outcome prediction, (3) Patient management plan, and (4) Assumption. All prompts and responses of ChatGPT as well as the graphical presentation of the performance, were documented in a Microsoft Excel spreadsheet.

## Results

**Figure 1:**
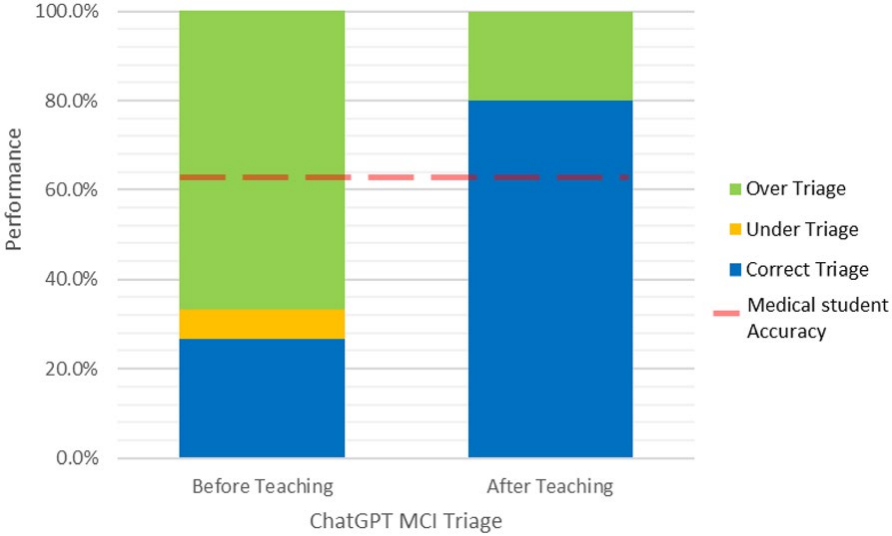
The bar graph depicts ChatGPT's performance in MCIs triage before (26.7%) and after (80%) instruction on the START triage algorithm. Additionally, a line graph shows the mean accuracy score of medical students on the same questionnaire (64.3%).

Our analysis found that before instruction on the START triage algorithm, the initial triage performance of ChatGPT was observed to yield an overall triage accuracy of 26.7% in correct triage, with 66.7% of cases being over-triaged and 6.7% of cases being under-triaged. After teaching the START triage algorithm, ChatGPT scored overall triage accuracy of 80% in correct triage, with 20% of cases being over-triaged and 0% being under-triaged. Figure 1 shows the overall performance of ChatGPT in comparison to medical student’s triage accuracy on the same MCIs triage questionnaire, with a mean accuracy score of 64.3%, published by Sapp et al. 2010^18^.

Our investigation utilized the thematic analysis method and highlighted the qualitative discoveries regarding ChatGPT's effectiveness before and after its instruction in the correct START triage algorithm. First, the major themes that emerged from these analyses were summarized. The subsequent content analysis section provided an overall presentation of the data.

In the context of MCIs, the *walking wounded* refers to individuals who have suffered minor injuries and can be safely transported to a designated casualty collection point for further assessment^25^. Before teaching the correct START algorithm, ChatGPT demonstrated an adept understanding of this theme, correctly identifying 13 out of 15 responses related to the walking wounded patients in the questionnaire. For example, in response to question(Q) 6 and 7:

Q6: ‘‘*…the patient is falling repeatedly, and unable to stand…*’’

Q7: ‘‘*…the patient is unresponsive and had a seizure…*’’

One of ChatGPT's responses on the walking wounded theme was deemed incorrect as it did not specify the victim's mobility status, despite the question clearly stating that this victim is able to aid others at the site of the MCI, as seen in Q1. It appeared that ChatGPT did not provide an accurate response to the question about the walking wounded. The question mentioned that the victim could not walk due to severe weakness, but this crucial detail was not addressed in ChatGPT's response, as seen in Q2.

After teaching the correct START algorithm, ChatGPT's performance on *the walking wounded* theme remains the same, accurately identifying 13 out of 15 responses. For example, ChatGPT got Q1 and Q2 correct.

Q1: ‘‘*…the patient appears alert and able to follow instructions …*’’

Q2: ‘‘*…The walking wounded patients are initially tagged as "green" or "minor." For the remaining victims, we would assess respirations first. In this case, the patient has a respiratory rate of 12, …*’’

Implying that the patient is unable to walk. Therefore proceed with the assessment of respiration.

However, ChatGPT answered Q3 and Q9 incorrectly. Specifically, ChatGPT failed to accurately identify the patient's mobility status in Q3, despite the scenario mentioning that the individual was running.

Q9: ‘‘*The walking patient with large cuts on the thigh after putting his leg through a glass door…*’’, despite clearly mentioned in the question that the patient is unable to stand.

The next theme was *respiration*. In START triage, the assessment of respirations is crucial in determining the appropriate triage category for patients who remain immobile^25^. Before teaching the correct START algorithm, ChatGPT demonstrated an adept understanding of this theme, accurately identifying 12 out of 15 responses related to the respiration of the patients in the questionnaire. For example, in response to Q2 and Q15:

Q2: ‘‘*…the respiratory rate is within normal limits …*’’

Q15: ‘‘*…since her respiratory rate is less than 30 breaths per minute… she began breathing spontaneously after the airway was opened…*’’

Implying that the patient has 0 respiratory rate that requires airway opening.

ChatGPT's incorrect responses in this section may be attributed to a lack of familiarity with the cut-off point for respiratory rate in accordance with the START triage algorithm. Per the guidelines, a respiratory rate greater than 30 is considered elevated^25^. However, ChatGPT incorrectly identified the respiratory rates in questions 3 and 4 as elevated, despite the rates being 24 and 26 breaths per minute, respectively.

After teaching the correct START algorithm, ChatGPT's performance on *the Respiration* theme remained the same, accurately identifying 12 out of 15 responses. Examples of mistakes made by ChatGPT included failing to mention the respiration rate of Q7 despite it being clearly stated in the question. Similar cases were also observed in Q9. ChatGPT also incorrectly quoted the number of respiratory rates, for example, in Q4.

Q4: ‘‘*…Respiratory rate is greater than 30, so the patient is classified as "red" or "immediate…*’’

However, the question clearly stated that the respiratory rate was 26.

The next theme was *perfusion*. In START triage, perfusion assessment can be done via radial pulse or capillary refill^25^. Before teaching the correct START algorithm, ChatGPT demonstrated a good understanding of this theme, accurately identifying 15 out of 15 responses related to the respiration of the patients in the questionnaire. For example, in response to Q4:

Q4: ‘‘*…although there is some bleeding, it is described as minor. The patient has a capillary refill time of less than 2 seconds, indicating good circulation, and a radial pulse is palpable …*’’

However, after teaching the correct START algorithm, ChatGPT’s performance on the Perfusion theme deteriorated, accurately identifying 14 out of 15 responses. The mistake can be seen in Q14.

Q14 ‘‘*…Cap refill is less than 2 seconds, indicating good perfusion, and she has a weak radial pulse …*’’

Although ChatGPT correctly identified that the cap refills less than 2 s, indicating good perfusion, the question did not mention ‘weak radial pulse.’ The question, however, mentioned ‘generalized weakness,’ which addressed muscle strength in medical terminology^29^, and no pulse. This finding suggests that ChatGPT is prone to mistakes with medical jargon. On the other hand, ChatGPT’s response to a similar question before the teaching of the START algorithm did not offer anything about perfusion, and it was considered correct because, according to the START algorithm, this patient can be triage without assessing the perfusion ‘minor’^25^.

The final pre-determined theme was about *mental status*. Patients with altered mental status should be classified as ‘immediate’ priority, as a head injury or other underlying emergency condition may contribute to the abnormal consciousness level^25^. ChatGPT displayed good comprehension of the theme, as evidenced by the perfect score of 15 out of 15 both before and after being taught the correct START algorithm. For example, in Q10, before and after teaching:

Q10 before teaching: ‘‘*…the patient is unresponsive in the driver's seat of a car at the hospital entrance …*’’

Q10 after teaching: ‘‘*…the patient is unresponsive……*’’

The overall content analysis of the pre-determined themes on ‘walking wounded,’ ‘respiration,’ ‘perfusion,’ and ‘mental status’ were shown in Table 1s. It presented almost similar performance on each theme before and after teaching the START algorithm except for the *perfusion* theme, with a performance of 93.3% before teaching and 86.7% after teaching the START algorithm.

**Table 1:** Content analysis of ChatGPT’s response on pre-determined themes before and after teaching the START algorithm.

	Content analysis before teaching		Content analysis after teaching	
Theme	Correct	Performance (%)	Correct	Performance (%)
*Walking wounded*	13/15	86.7	13/15	86.7
*Respiration*	12/15	80.0	12/15	80.0
*Perfusion*	14/15	93.3	13/15	86.7
*Mental Status*	15/15	100	15/15	100

Our in-depth qualitative analysis of ChatGPT’s response to the mass casualty incident triage questionnaire revealed an additional theme and subtheme. For a summary of the theme and sub-theme, please see Table 2. For examples and details relating to each theme, please see Supplementary Table S3 and S4.

**Table 2:** Additional themes and subthemes identified from ChatGPT’s response before and after teaching the START algorithm.

Additional theme identified before teaching START Algorithm		
Disclaimer	Prediction	Management plan
Requires professional medical advice	Etiology prediction Clinical outcome prediction	Urgency Logistic plan Clinical investigation

Additional theme identified After teaching START Algorithm		
Assumption	Prediction	Management plan
Inferential	Etiology prediction Clinical outcome prediction	Urgency Logistic plan Clinical investigation

### Disclaimer

#### Requires professional medical advice

In response to the MCIs questionnaire, ChatGPT, in many instances, emphasized that the presented scenario is hypothetical in nature and that any actual triage decisions should be made exclusively by trained medical professionals. In addition, ChatGPT highlighted that evaluation by medical professionals is necessary to provide appropriate management.

‘‘*…It's important to note that this is a hypothetical scenario, and that actual triage decisions should be made by a trained medical professional who has access to the patient and can conduct a thorough evaluation of their condition*.’’

### Prediction

#### Etiology prediction

In response to the MCIs questionnaire, ChatGPT made several predictions regarding the injuries and medical conditions of the patient, despite the absence of any information regarding the patient's ultimate outcome. One of those predictions were etiology prediction. In our case, *etiology* is defined as the causation or origin of trauma and medical conditions. For example:

‘‘*…The cause of the patient's confusion, falls, and inability to stand is not clear from the information provided, but this could be due to a variety of conditions such as a head injury, stroke, or seizure, among others…*’’

‘‘…*The presence of a bruise on the forehead also raises the possibility of a head injury, which further increases the urgency of care..*.’’

#### Clinical outcome prediction

In addition to predicting the etiology of injuries and medical conditions in response to the MCIs questionnaire, ChatGPT also provided clinical outcome predictions, such as predicting morbidity and mortality of the patients if urgent medical attention is unavailable. In other cases, ChatGPT predicted that the patient with symptoms requiring urgent medical attention could have been initially wrongly tagged as ‘minor’ since she could walk. For example:

‘‘*… The patient is critically ill and requires immediate medical attention to prevent permanent brain damage or death.*’’

‘‘*…she can be directed to a casualty collection point and tagged as "green" or "minor" initially… The patient is alert but exhibiting symptoms such as chest tightness, blurry vision, drooling, and weakness. These symptoms could indicate a serious underlying condition and warrant immediate medical attention. Therefore, the patient should be re-triaged and tagged as "red" or "immediate" for further evaluation and treatment.*’’

### Management plan

#### Urgency

Although the START triage algorithm does not include the patient's management plan, ChatGPT's responses highlighted the urgent medical attention for the patient in the MCIs questionnaire. These recommendations prioritize the urgency of the patient's needs, highlighting the importance of timely and appropriate intervention. For example:

‘‘*…Patient's injuries require immediate medical attention to control bleeding, prevent infection, and ensure adequate circulation…*’’

‘‘*The patient should be treated with emergency interventions, such as oxygen therapy, medications to manage the underlying condition, and possibly intubation or other airway management interventions…*’’

#### Logistic plan

Apart from that, ChatGPT also planned for the logistical requirements of patient transportation to hospitals or medical facilities, despite needing to be included in the START triage algorithm. This logistic consideration reflects a crucial aspect of patient management, ensuring that the patient receives timely and appropriate care. For example:

‘‘…*The patient should be transported to the hospital as soon as possible for further evaluation and treatment…*’’

‘‘…*The patient should be transported to the hospital for further evaluation and treatment, but it is not urgent…*’’

#### Clinical investigation

Furthermore, ChatGPT provided recommendation on clinical investigation for some patients, which were not included in the START triage algorithm. Despite this deviation from the algorithm, such a suggestion can aid medical personnel in managing and diagnosing the patient. For example:

‘‘… *The patient may require imaging studies (such as CT scan or MRI) and interventions to manage the underlying condition, such as medications to manage blood pressure or surgery to treat bleeding in the brain…*’’

#### Inferential assumption

Lastly, in many cases, ChatGPT extrapolated and made inferential reasoning based on the incomplete or limited information that needed to be provided. For example:

‘‘*…The walking patient with large cuts on the thigh after putting his leg through a glass door … Since there is no information given about his breathing, we assume that he is breathing…*’’

## Discussion

Our findings showed that after receiving instruction on START triage, ChatGPT demonstrated a higher level of performance in MCI triage scenarios compared to medical students, physicians, registered nurses, and paramedics^18,30^. These findings are supported by previous research, which has consistently indicated the superiority of AI-based tools over healthcare professionals^12,31–33^. For example, a recent study documented that ChatGPT provides significantly higher quality and empathetic responses to patients' questions compared to physician responses (t = 13.3; p < 0.001)^31^. Similarly, ChatGPT also performed close to the passing cut points for all three exams of the United States Medical Licensing Exam (USMLE) without any training^34^. Another study by Levin et al.^35^ argued that an AI-based electronic triage tool performs equivalent to or better than the US Emergency Severity Index (ESI). Furthermore, Yu et al.^36^ showed that machine and deep learning-based triage in ED predicts clinical outcomes more correctly than existing triage systems.

Nevertheless, a plausible explanation for the higher performance of AI tools compared to human workers is their capacity to concurrently handle multiple variables by leveraging extensive datasets for predicting complex outcomes^37^. Besides, AI can reduce metacognitive errors and illusory correlations in emergency medicine (i.e., diagnosis of sepsis)^12,38^, while human decisions generally mix with potential biases and heuristics^32,39^. Therefore, while AI tools cannot substitute human cognition, interventions based on AI hold significant promise in enhancing emergency and disaster medicine, clinical decision-making, and medical education^12,40^. However, further research is imperative prior to their clinical implementation.

The predetermined content analysis conducted on ChatGPT's performance, both before and after teaching START triage, revealed no significant variation in scores across the themes of "walking wounded," "respiration," "perfusion," and "mental status.". These findings demonstrate that ChatGPT possesses the capability to comprehend the information presented in the MCIs questionnaire, including the comprehension of medical abbreviations. Nevertheless, a significant improvement was observed in ChatGPT's overall triage performance after teaching the START algorithm. These findings indicate that before instruction on START algorithms, ChatGPT exhibited accurate processing of predetermined thematic information but did not effectively apply the START triage algorithm to achieve the final overall triage outcome.

Thematic analysis of ChatGPT's responses to the MCIs questionnaire further revealed additional themes: medical disclaimers, etiology and clinical outcome prediction, management plans to encompass urgency, logistics, clinical investigations, and inferential assumptions. These findings offered a glimpse into the potential of AI to support decision-making for first responders during times of catastrophes and disasters, mainly when human resources are scarce. First responders face challenges due to fatigue-related neurocognitive and physical performance decrements^41^.

While ChatGPT was not explicitly designed for mass casualty triage, its remarkable performance underscores its significant potential. Nevertheless, as ChatGPT is a language-based AI, further research is warranted to explore its applicability in clinical or real-world MCIs, particularly in translating patients' vital signs into interpretable information for AI systems. It is essential to acknowledge that certain limitations exist when employing AI during MCIs or disasters, including challenges related to power supply, internet availability, and the affordability of such technology.

Although AI tools like ChatGPT have shown great promise in healthcare and emergency medicine, and the field is developing rapidly, it raises an excessive concern for healthcare systems, patients, society, and bioethical questions^6,42^. Additionally, researchers, policymakers, and the general public worry about privacy, security, equitable access, clinical safety, and accountability with risk and benefit assessment of AI tools^6^. For example, ChatGPT gives false information, and doubtful and inconsistent advice sometimes^3,43^, and Italy already banned ChatGPT for privacy concerns^44^. Even though, human judgment seems to be inconsistent, ensuring consistency is a crucial undisputable ethical issue. Actually, human judgment is often constructed on intuition instead of reason and has a higher likelihood of being sensitive to biases, emotions, and fallacies^45–47^.

Conversely, AI bots have no emotions like a human, therefore they are used as an assistant to facilitate increasing human judgment^48^. In addition, in this current early stage, AI lacks the capability to detect unforeseen hazards, such as the scent of leaking fuel during a major road accident or making triage choices during severe weather events like heavy snowstorms. In future, there might be sensors that transform environmental data into a real-time format 'comprehensible to AI' for evaluation. Nevertheless, even with these advancements, they cannot replace the hands-on clinical experience and innate intuition of a first responder.

Moreover, unintended outcomes for patients may appear due to hacking of the system^49,50^ and it is also highly challenging to verify the AI-related intervention due to the scarcity of immediately available peer-reviewed studies and the interdisciplinary nature of the field^51^. As a result, contributors of AI tools must maintain ethical requirements when developing and releasing any responsible AI tools^42,52^, and more research is required on how to address the existing bioethical, clinical, and technical limitations of medical AI.

Future research examining AI-chatbot efficacy should emphasize evaluating the precision of different AI algorithms in conducting disaster triage and their capability to reduce under-triage. Other critical considerations encompass the practicality of these systems, their response speed, and a thorough cost–benefit assessment. An important aspect is the incorporation of sensor technology, which can offer real-time data collection on patients during a MCI (like vital statistics), the incident environment (including potential hazards at the scene), and the available capacity such as response resources coordination, communication channels, and prioritization strategies.

## Conclusion

ChatGPT showed promising results in effectively responding to MCIs questionnaires, highlighting its potential to assist in situations where human resources are scarce during such incidents. Nevertheless, additional research is required to ensure its safety and efficacy before clinical implementation.

### Supplementary Information

Supplementary Information.

## Data Availability

The datasets generated during and/or analyzed during the current study are available from the corresponding author on reasonable request. Additionally, medical students' triage performance data are accessible from the following link https://doi.org/10.1017/S1049023X00008104.

## References

[1] Analysis | Is ChatGPT the Start of the AI Revolution? *Washington Post* (2022).

[2] Is ChatGPT the Start of the AI Revolution? - Bloomberg. https://www.bloomberg.com/opinion/articles/2022-12-09/is-chatgpt-the-start-of-the-ai-revolution#xj4y7vzkg?leadSource=uverify wall.

[3] Krügel S, Ostermaier A, Uhl M (2023). ChatGPT’s inconsistent moral advice influences users’ judgment. Sci. Rep..

[4] Heilweil, R. AI is finally good at stuff. Now what? *Vox* https://www.vox.com/recode/2022/12/7/23498694/ai-artificial-intelligence-chat-gpt-openai (2022).

[5] EIT Health and McKinsey & Company. Transforming healthcare with AI-The impact on the workforce and organisations. (2020).

[6] Lekadir, K., Quaglio, G., Garmendia, A. T. & Gallin, C. Artificial intelligence in healthcare applications, risks, and ethical and societal impacts. *EPRS Eur. Parliam. Res. Serv.*10.2861/568473 (2022).

[7] Xia M, Xu T, Jiang H (2022). Progress and perspective of artificial intelligence and machine learning of prediction in anesthesiology. J. Shanghai Jiaotong Univ. Sci..

[8] Briganti G, Pham TD, Yan H, Ashraf MW, Sjöberg F (2021). Augmented medicine: Changing clinical practice with artificial intelligence. Advances in Artificial Intelligence, Computation, and Data Science: For Medicine and Life Science.

[9] Briganti G, Le Moine O (2020). Artificial intelligence in medicine: Today and tomorrow. Front. Med..

[10] Biswas SS (2023). Role of chat GPT in public health. Ann. Biomed. Eng..

[11] Gómez-González, E. & Gómez Gutiérrez, E. Artificial intelligence in medicine and healthcare: Applications, availability and societal impact. 10.2760/047666 (2020).

[12] Kirubarajan A, Taher A, Khan S, Masood S (2020). Artificial intelligence in emergency medicine: A scoping review. J. Am. Coll. Emerg. Phys. Open.

[13] Berlyand Y (2018). How artificial intelligence could transform emergency department operations. Am. J. Emerg. Med..

[14] Greaves I, Dyer P, Porter KM (1995). Handbook of Immediate Care.

[15] Smith W (2012). Triage in mass casualty situations. CME Your SA J. CPD.

[16] Bazyar, J., Farrokhi, M. & Khankeh, H. Triage Systems in Mass Casualty Incidents and Disasters: A Review Study with A Worldwide Approach. **7**, 482–494. 10.3889/oamjms.2019.119 (2019). 10.3889/oamjms.2019.119 PMC6390156 30834023

[17] Kennedy K, Aghababian RV, Gans L, Lewis CP (1996). Triage: Techniques and applications in decision making. Ann. Emerg. Med..

[18] Sapp RF, Brice JH, Myers JB, Hinchey P (2010). Triage performance of first-year medical students using a multiple-casualty scenario, paper exercise. Prehosp. Disaster Med..

[19] Kilner T (2002). Triage decisions of prehospital emergency health care providers, using a multiple casualty scenario paper exercise. Emerg. Med. J..

[20] Bergeron S, Gouin S, Bailey B, Patel H (2002). Comparison of triage assessments among pediatric registered nurses and pediatric emergency physicians. Acad. Emerg. Med..

[21] Lu S, Christie GA, Nguyen TT, Freeman JD, Hsu EB (2022). Applications of artificial intelligence and machine learning in disasters and public health emergencies. Disaster Med. Public Health Prep..

[22] Tang P, Shen GQ (2015). Decision-making model to generate novel emergency response plans for improving coordination during large-scale emergencies. Knowl. Based Syst..

[23] Kim D (2018). A data-driven artificial intelligence model for remote triage in the prehospital environment. PLoS ONE.

[24] Natalie. ChatGPT—Release Notes | OpenAI Help Center. https://help.openai.com/en/articles/6825453-chatgpt-release-notes.

[25] START Adult Triage Algorithm - CHEMM. https://chemm.hhs.gov/startadult.htm.

[26] Mohd Sulaiman I, Bulgiba A, Abdul Kareem S (2022). Prevalence and risk factors for dangerous abbreviations in Malaysian electronic clinical notes. Eval. Health Prof..

[27] Tariq RA, Sharma S (2022). Inappropriate Medical Abbreviations.

[28] Peng J, Xiang H (2016). Trauma undertriage and overtriage rates: Are we using the wrong formulas?. Am. J. Emerg. Med..

[29] DeLuca, J. & Barrett, A. M. Weakness and Fatigue. *Imaging Acute Neurol. Dis. Symptom-Based Approach* 347–358 (1990). 10.1017/CBO9781139565653.023.

[30] Risavi BL, Salen PN, Heller MB, Arcona S (2009). A two-hour intervention using start improves prehospital triage of mass casualty incidents. Prehosp. Emerg. Care.

[31] Ayers JW (2023). Comparing physician and artificial intelligence chatbot responses to patient questions posted to a public social media forum. JAMA Intern. Med..

[32] Krittanawong C (2018). The rise of artificial intelligence and the uncertain future for physicians. Eur. J. Intern. Med..

[33] Li, J., Dada, A., Kleesiek, J. & Egger, J. ChatGPT in Healthcare: A Taxonomy and Systematic Review. 2023.03.30.23287899. Preprint at 10.1101/2023.03.30.23287899 (2023).

[34] Kung TH (2023). Performance of ChatGPT on USMLE: Potential for AI-assisted medical education using large language models. PLoS Digit. Health.

[35] Levin S (2018). Machine-learning-based electronic triage more accurately differentiates patients with respect to clinical outcomes compared with the emergency severity index. Ann. Emerg. Med..

[36] Yu JY, Jeong GY, Jeong OS, Chang DK, Cha WC (2020). Machine learning and initial nursing assessment-based triage system for emergency department. Healthc. Inform. Res..

[37] Tang KJW (2021). Artificial intelligence and machine learning in emergency medicine. Biocybern. Biomed. Eng..

[38] Schinkel M, Paranjape K, Nannan Panday RS, Skyttberg N, Nanayakkara PWB (2019). Clinical applications of artificial intelligence in sepsis: A narrative review. Comput. Biol. Med..

[39] Beam AL, Kohane IS (2016). Translating artificial intelligence into clinical care. JAMA.

[40] Mbakwe AB, Lourentzou I, Celi LA, Mechanic OJ, Dagan A (2023). ChatGPT passing USMLE shines a spotlight on the flaws of medical education. PLoS Digit. Health.

[41] Yung M, Du B, Gruber J, Hackney A, Yazdani A (2022). Fatigue measures and risk assessment tools for first responder fatigue risk management: A scoping review with considerations of the multidimensionality of fatigue. Saf. Sci..

[42] Much to discuss in AI ethics. *Nat. Mach. Intell.* **4**, 1055–1056 (2022).

[43] Borji, A. A categorical archive of ChatGPT failures. *ArXiv Prepr. *arXiv:2302.03494 (2023).

[44] Kreitmeir, D. H. & Raschky, P. A. The Unintended Consequences of Censoring Digital Technology--Evidence from Italy’s ChatGPT Ban. *ArXiv Prepr. *arXiv:2304.09339 (2023).

[45] Haidt J (2001). The emotional dog and its rational tail: A social intuitionist approach to moral judgment. Psychol. Rev..

[46] Greene JD, Sommerville RB, Nystrom LE, Darley JM, Cohen JD (2001). An fMRI investigation of emotional engagement in moral judgment. Science.

[47] Greene JD, Morelli SA, Lowenberg K, Nystrom LE, Cohen JD (2008). Cognitive load selectively interferes with utilitarian moral judgment. Cognition.

[48] Lara F, Deckers J (2020). Artificial intelligence as a socratic assistant for moral enhancement. Neuroethics.

[49] Amodei, D. *et al.* Concrete problems in AI safety. *ArXiv Prepr. *arXiv:1606.06565 (2016).

[50] Challen R (2019). Artificial intelligence, bias and clinical safety. BMJ Qual. Saf..

[51] Paranjape K, Schinkel M, Panday RN, Car J, Nanayakkara P (2019). Introducing artificial intelligence training in medical education. JMIR Med. Educ..

[52] Prunkl CE (2021). Institutionalizing ethics in AI through broader impact requirements. Nat. Mach. Intell..

