# Supplementary material for: ChatGPT's performance before and after teaching in mass casualty incident triage
Source: Sci Rep. 2023 Nov 21;13:20350. doi: 10.1038/s41598-023-46986-0 (PMC10663620; doi:10.1038/s41598-023-46986-0)
Supplement: Supplementary file 1 — Supplementary Information. [file 41598_2023_46986_MOESM1_ESM.docx]

**Supplementary Tables**

**Supplementary Table S1**: Mass casualty incident triage questionnaire by Sapp et al. utilized in this research, reproduced with written permission.

| **Triage Scenarios** | | | | |
| --- | --- | --- | --- | --- |
| R | Y | G | B | 25 y/o male appears alert c/o headache and cough after assisting someone from scene. Denies trauma. R 18 |
| R | Y | G | B | 32 y/o c/o is unable to walk due to severe weakness, sweating and uncontrolled diarrhea. No visible trauma. Cap refill <2 sec, R 12 |
| R | Y | G | B | 33 y/o male asthmatic alert c/o shortness of breath after running from scene. Vital signs R 24 |
| R | Y | G | B | 34 y/o female alert c/o severe R leg pain with a fractured lower leg with bone sticking out of the skin after being pushed down approx 6 stairs. Bleeding appears to be minor. Denies other complaint. Cap refill <2 sec, radial pulse palpable. R 26. |
| R | Y | G | B | 38 y/o confused male who initially c/o severe shortness of breath and crushing chest pain but became unresponsive in the ambulance. CPR in progress by EMS on arrival at hospital. |
| R | Y | G | B | 40 y/o female is confused, falling repeatedly and unable to stand. Is unable to follow simple commands. Cap refill <2sec, good radial pulse. R 14 |
| R | Y | G | B | 44 y/o male having seizure that stops on arrival at the hospital. He is unresponsive, There is a bruise on his forehead. Cap refill <2 sec, good radial pulse. R 14 |
| R | Y | G | B | 45 y/o male is walking alert c/o right wrist pain after fall. Evident deformity of R wrist. No visible bleeding. Vital signs R 20 |
| R | Y | G | B | 48y/o alert male with large cuts on thigh after putting his leg through a glass door. He is unable to stand. There is visible muscle and tendon with controllable bleeding. Cap refill <2 sec, good radial pulse. R 18 |
| R | Y | G | B | 51 y/o male unresponsive in drivers seat of car at hospital entrance. He has no visible trauma. His face is blue and he is not breathing. Cap refill >2 sec, barely palpable radial pulse. R O after opening his airway. |
| R | Y | G | B | 55 y/o alert male c/o headache, slurred speech and blurry vision. Falls repeatedly on standing but has no visible trauma. Cap refill < 2 sec, good radial pulse. R 14. |
| R | Y | G | B | 56 y/o female alert, c/o shortness of breath, chest tightness, blurry vision, drooling and weakness. Cap refill <2 sec, good radial pulse. R 36 |
| R | Y | G | B | 56 y/o female unresponsive according to EMS she, “Just stopped breathing!” No visible trauma. Cap refill <2 sec, weak radial pulse. R 0 after opening her airway. |
| R | Y | G | B | 60 y/o female police officer presents after driving herself to hospital. She was the first arriving officer and aided the wounded. She is alert c/o generalized weakness, wet with sweat, and drooling. No visible trauma R 14 |
| R | Y | G | B | 63 y/o female face down unresponsive. Her face is blue and she does not appear to be breathing. There is no visible trauma. Cap refill <2 sec, good radial pulse. R 0, Begins breathing spontaneously after opening her airway. |

**Supplementary Table S2:** Prompt applied to teach ChatGPT the correct START triage steps and clarify the medical abbreviation from the triage questionnaire.

| **Prompt A : Prompt used to teach ChatGPT the correct flow of START triage** (59,60)**.**  The followings are the correct algorithm for START triage.  First step, prioritize those with the most urgent medical needs. This can be achieved by instructing individuals with minor injuries or those deemed to have a low risk of imminent death to relocate to a designated casualty collection point. Using a loud voice, such instructions can be communicated through the help of a public address system or loudspeaker. Patients who are capable of walking, referred to as the "walking wounded," are initially categorized as "green" or "minor" during triage. While it is possible that these patients may have severe injuries, their ability to comprehend instructions, move independently, and reach a designated casualty collection point indicates a low likelihood of immediate mortality. However, it is imperative to conduct a re-triage of these patients, including the walking wounded, to detect any underlying serious conditions that may have been overlooked during the initial assessment.  Remaining victims may fall into categories such as those who are unable to comprehend instructions, unable to move, unconscious, or deceased. The next step is to evaluate their breathing.  The assessment of respirations is crucial in determining the appropriate triage category for patients who remain. Repositioning the airway may be necessary for those who are not breathing, but if breathing does not commence spontaneously, the patient is considered "deceased." If the patient begins breathing following airway repositioning, placement of an oral airway is recommended, and they are classified as "immediate" priority. A respiratory rate exceeding 30 breaths per minute indicates a "red" or "immediate" priority due to potential shock or respiratory distress.  Next, assess perfusion.  In patients with a respiratory rate of less than 30 breaths per minute, the subsequent step is to evaluate perfusion or circulation status via radial pulse or capillary refill. A missing radial pulse or capillary refill more than 2 seconds warrants an "immediate" priority classification. Since breathing is present, the presence of a heartbeat is presumed, as cessation of breathing would ensue if no heartbeat was present for an extended period.  Next, assess mental status.  The final assessment parameter is the mental status of the patient. Patients with unimpaired mental status are designated as "yellow" or "delayed." These individuals may be immobile due to lower extremity injuries or other factors that impeded their ability to reach the "green" area. However, if a patient displays confusion or an altered mental state, they are classified as "immediate" priority, as a head injury or other underlying condition may be contributing to the abnormal consciousness level. |
| --- |
| **Prompt B: Prompt applied to clarify all medical abbreviation used in triage questionnaire**  Clarification for short forms:  y/o= Years Old,  R= respiratory rate per minutes,  R= right side,  Cap refill= capillary refills time in seconds,  c/o= complaints of. |

**Supplementary Table S3:** Additional theme identified before teaching START algorithm.

| Theme | Subthemes | ChatGPT Quotes |
| --- | --- | --- |
| Disclaimer | Requires professional medical advice | …It's important to note that this is a hypothetical scenario, and that actual triage decisions should be made by a trained medical professional who has access to the patient and can conduct a thorough evaluation of their condition. |
|  |  | … A thorough evaluation by a medical professional is necessary to determine the cause and provide appropriate treatment. |
|  |  | … Further evaluation by a medical professional is necessary to determine the extent of injury and provide appropriate treatment. |
| Prediction | Clinical outcome prediction | … The patient is exhibiting severe weakness, sweating, and uncontrolled diarrhea. These symptoms could be indicative of a serious medical condition... |
|  |  | …However, the vital signs do not suggest immediate danger… |
|  |  | … The patient's injuries are severe and life-threatening. The patient's inability to stand may indicate further injuries. |
|  |  | … The patient is critically ill and requires immediate medical attention to prevent permanent brain damage or death. |
|  |  | …Indicating that she may be in shock or experiencing some other type of medical emergency. |
|  |  | … the patient appears to be experiencing symptoms that may indicate a medical emergency. |
|  |  | … may suggest that she has been exposed to a hazardous environment, which could further increase her risk of injury or illness |
|  | Etiology | … The respiratory rate of 26 is elevated, but could be due to pain or anxiety. |
|  |  | … The cause of the patient's confusion, falls, and inability to stand is not clear from the information provided, but this could be due to a variety of conditions such as a head injury, stroke, or seizure, among others. |
|  |  | …The cause of the patient's seizure and unresponsiveness is not clear from the information provided, but this could be due to a variety of conditions such as head injury, stroke, or seizure disorder, among others |
|  |  | … It's important to note that the patient has an evident deformity of the wrist, which indicates a possible fracture. |
|  |  | … The patient's lack of breathing and poor circulation suggest that he is in cardiac arrest or respiratory arrest |
|  |  | …The patient's symptoms suggest a neurological problem, such as a stroke or a brain injury. |
|  |  | … The patient's symptoms suggest a potentially life-threatening condition, such as a heart attack or a severe allergic reaction (anaphylaxis) |
|  |  | … which may be signs of a serious medical condition such as a stroke or heart attack. |
|  |  | … her airway appears to have been obstructed. |
|  |  | … This may be due to a variety of causes, such as choking, a foreign object in the airway, or an allergic reaction. |
| Management plan | Urgency | Patient's injuries require immediate medical attention to control bleeding, prevent infection, and ensure adequate circulation. |
|  |  | But immediate intervention with advanced life support measures, such as chest compressions, defibrillation, and advanced airway management, is necessary to resuscitate the patient. |
|  |  | The patient should be treated with emergency interventions, such as oxygen therapy, medications to manage the underlying condition, and possibly intubation or other airway management interventions. |
|  |  | Further underscores the need for immediate intervention to manage her airway and support her breathing. |
|  |  | …prompt medical evaluation and treatment are necessary to ensure her safety and well-being. |
|  |  | … requires immediate medical evaluation and treatment to determine the cause of her episode and ensure that she does not experience any further complications. |
|  | Logistic plan | The patient should be transported to the hospital for further evaluation and treatment, but it is not urgent. |
|  |  | The patient should be transported to the hospital as soon as possible for further evaluation and treatment. |
|  | Clinical investigations | … The patient may require imaging studies (such as CT scan or MRI) and interventions to manage the underlying condition, such as medications to manage blood pressure or surgery to treat bleeding in the brain. |

**Supplementary Table S4**: Additional theme identified after teaching START Algorithm

| Theme | Subthemes | ChatGPT Quotes |
| --- | --- | --- |
| Predictive | Clinical outcome prediction | …this patient should be tagged as "Immediate" and should be quickly evaluated by a medical professional as she has an altered mental status and is at risk for worsening or deteriorating. |
|  |  | However, there is no indication of altered mental status or confusion, so we would tag him as "yellow" or "delayed" since he has a potentially serious injury… but is not immediately life-threatening.. |
|  |  | She can be directed to a casualty collection point and tagged as "green" or "minor" initially…… the patient should be re-triaged and tagged as "red" or "immediate" for further evaluation and treatment. |
|  |  | …Indicating that they have no chance of survival without immediate medical intervention… |
|  |  | … her symptoms of generalized weakness, sweating, and drooling may be indicative of a medical emergency… |
|  | Etiology | The presence of a bruise on the forehead also raises the possibility of a head injury, which further increases the urgency of care. |
|  |  | However, the symptoms of headache, slurred speech, and blurry vision, as well as repeated falls, indicate a potential neurological or cardiovascular issue... |
| Management plan | Urgency | However, further assessment of the patient's perfusion and mental status is necessary to determine if their condition warrants re-triaging to a higher priority category |
|  |  | He requires immediate medical attention to determine the cause of his seizure and level of consciousness, and to initiate appropriate treatment. |
|  |  | The patient should be monitored closely for any changes in his condition and treated as soon as possible. |
|  |  | His large cuts on the thigh with visible muscle and tendon require immediate medical attention, but his vital signs indicate that he is not in immediate danger of death. |
|  |  | The focus should shift to providing immediate medical intervention to attempt resuscitation. |
|  |  | The patient is alert but exhibiting symptoms such as chest tightness, blurry vision, drooling, and weakness. These symptoms could indicate a serious underlying condition and warrant immediate medical attention. |
|  |  | …she may be re-triaged as "delayed" or "minor" once she is fully assessed and treated. |
|  | Logistic plan | …and transported to a medical facility for further evaluation and treatment. |
|  | Clinical investigations plan | So she should be further evaluated by a medical professional to determine the underlying cause of her symptoms. |
| Assumption | Inferential | …The walking patient with large cuts on the thigh after putting his leg through a glass door … Since there is no information given about his breathing, we assume that he is breathing… |
|  |  | The patient is currently receiving CPR, which indicates immediate or emergent need for medical attention… |
